# Outcomes of the Supine Anterior-based Muscle-sparing Approach for Primary and Revision Hip Arthroplasty

**DOI:** 10.5435/JAAOSGlobal-D-21-00050

**Published:** 2022-02-02

**Authors:** Tommy Pan, Anuj Mehta, Mark W. Mason

**Affiliations:** From the Penn State College of Medicine, Hershey, PA (Mr. Pan, Mr. Mehta, and Dr. Mason), and Penn State Hershey Medical Center, Bone and Joint Institute (Dr. Mason).

## Abstract

Risks and outcomes of total hip arthroplasty (THA) are believed to vary relative to the surgical approach. This study compares the supine anterior-based muscle-sparing (ABMS) approach with its modern-day counterparts.

**Methods:**

A retrospective review was done on 550 patients undergoing primary or revision THA from 2016 to 2018. Surgical modalities included direct anterior (DAA), ABMS, posterolateral, and Müller modified Hardinge approaches. Surgical data were collected, and clinical outcomes were measured by the Hip Disability and Osteoarthritis Outcome Score, Modified Harris Hip Score, UCLA, and VR-12 Mental/Physical scores preoperatively and compared clinical outcomes among approaches.

**Results:**

A total of 550 patients were included (447 primaries, 103 revisions). The average age was 64 years (231 men, 319 women). Approaches included 79 DAA (14%), 212 ABMS (39%), 180 modified Müller-Hardinge (33%), and 79 posterolateral (14%). The incidence of lateral femoral cutaneous nerve injury was more common with the DAA (*P* = 0.008), but no other clinically significant differences were noted among the groups.

**Conclusion:**

The results of this study showed no clinically notable differences between the supine ABMS and other approaches. The supine ABMS approach is an acceptable approach in modern day THA when used by an experienced surgeon well-versed in the approach.

Total hip arthroplasty (THA) is considered one of the most effective orthopaedic procedures for relieving pain, restoring function, and improving the quality of life in patients with hip osteoarthritis. Modern improvements in THA have yielded shorter hospital stays, better functional outcomes, and higher patient satisfaction scores.^1-3^ Although some suggest that a surgical approach is an independent predictor of an early postoperative outcome, it remains controversial whether one approach is superior to another.^4,5,6,7^

In 2004, Bertin and Röttinger^8^ described a minimally invasive hip approach using the standard Watson-Jones interval, which is the intermuscular plane between the tensor fascia lata and gluteus medius (GM) muscles. More recently labeled the anterior-based muscle-sparing (ABMS) approach, it has risen in popularity because of its reported early functional recovery and excellent clinical results.^4,9,10,11,12,13,14,15,16^ Although the ABMS approach is believed to reduce the risk of sciatic nerve injury and dislocation rates, it can damage the GM muscle during manipulation, causing abductor weakness.^8,9^ The ABMS approach can be successfully done in both the lateral^8,9,11-13,16,17^ and supine positions.^5,10,15,17^ However, it remains unclear whether the supine ABMS approach offers any advantage over other approaches. In addition, little has been written regarding the use of the ABMS approach for revision surgery.^18^ The purpose of this study was to review early postoperative outcomes of the supine ABMS approach compared with the direct anterior approach (DAA), modified Müller-Hardinge (MMH), and posterolateral (PL) approaches at a single institution for both primary and revision hip arthroplasties.

## Methods

This study was approved by the College of Medicine Institutional Review Board. A retrospective chart review was conducted on 550 consecutive patients who underwent primary or revision THA between 2016 and 2018. Four surgical approaches were used: DAA, supine modified Watson-Jones (ABMS), PL, and lateral MMH. Series of consecutive patients using each approach during the given period were selected for review. Five joint arthroplasty surgeons, each performing at least 100 primary arthroplasties a year at the time of this study, contributed cases to the series. All procedures in this study were done at a university academic tertiary referral center. The DAA procedures were all done on a HANA SC Mizuho OSI^19^ table, the MMH and PL approaches were done in the lateral decubitus position on a standard table, and the supine ABMS approach was done on a regular operating room table. The incision for the supine ABMS approach is similar to that of the MMH approach in that it is centered over the lateral aspect of the femur. The proximal portion of the incision veers slightly anterior compared with the MMH incision but is generally 5 to 10 cm more posterior than the DAA incision. The longitudinal division of the tensor fascia lata 1 to 2 cm posterior to the insertion of the tensor fascia muscle provides access to the interval between the GM and the tensor fascia.

For each patient, the data extracted included age, sex, laterality, body mass index (BMI), smoking history, chronic medical conditions (diabetes, hypertension, chronic kidney disease, and/or immunocompromised), primary reason for arthroplasty, surgical approach, anesthesia type (general, spinal, and/or periarticular), length of operation (LOO), estimated volume of blood loss (EBL), units of blood transfusion, complication type, and length of hospital stay (LOS). The institutional general indication for postoperative blood transfusion is symptomatic low hemoglobin that does not respond to adequate fluid resuscitation. Indications for specific individual's transfusions were not recorded. Clinical outcomes were measured by the Hip Disability Osteoarthritis Outcome Scores (HOOSs), UCLA, VR-12 Mental/Physical, Modified Harris Hip Score (HHS), and patient-reported outcome scores at both preoperative and 1-year follow-up.

Baseline characteristics and demographics were summarized for each approach using descriptive statistics (means, SDs, frequencies, and percentages). Means of continuous baseline measures were compared among approaches by analysis of variance, and distributions of categorical measures were compared by chi-square tests. The distribution of each outcome measure was evaluated to determine the best approach for analysis, either applying the natural log transformation or categorizing the ordinal measures where appropriate. Continuous outcomes were compared among the approaches with adjustment for important baseline characteristics by analysis of covariance, and the results reported for overall *P* values, model-adjusted means, and 95% confidence intervals (CIs). Binary outcomes were compared by logistic regression models with adjustment for important baseline characteristics, and the results reported for overall *P* values, adjusted odds ratios, and 95% CI. Significance was defined as *P* < 0.05. It was hypothesized that there would be no notable difference regarding in-hospital findings, perioperative complications, and 1-year functional outcome scores in the ABMS approach compared with its contemporary counterparts.

## Results

A total of 550 patients were included for review (329 right, 221 left). The cohort baseline characteristics and demographics are provided in Table 1. The average age was 63.7 years (range, 19-94 years). Two hundred thirty-one were men, and 319 were women. The average BMI was 30.8 (range, 16-48). The average number of chronic medical conditions was 1.0 (range, 0-3) and included a history of smoking (239), diabetes (84), hypertension (360), chronic kidney disease (23), and immunocompromised (87).

**Table 1:** Cohort Baseline Characteristics and Demographics

Characteristic	ABMS (n = 212, 38.6%)	DAA (n = 79, 14.4%)	MMH (n = 180, 32.7%)	POST (n = 79, 14.4%)	*P* Value
Age	60.6 (13.2)	62.4 (13.3)	66.6 (12.8)	67.1 (11.6)	<0.001
BMI	30.5 (6.2)	28.5 (5.1)	31.8 (6.0)	32.0 (7.1)	<0.001
Sex (M)	96 (45.3%)	26 (32.9%)	73 (40.6%)	36 (45.6%)	0.24
Smoking history	100 (47.2%)	41 (51.9%)	61 (33.9%)	37 (47.4%)	0.014
Diabetes	32 (15.1%)	5 (6.3%)	27 (15.0%)	9 (13.2%)	0.23
HTN	139 (65.6%)	43 (54.4%)	124 (68.9%)	54 (68.4%)	0.14
CKD	12 (5.7%)	4 (5.1%)	3 (1.7%)	4 (5.1%)	0.23
Immunosuppression	41 (19.3%)	10 (12.7%)	26 (14.4%)	10 (12.7%)	0.33
Blood thinners	6 (2.9%)	0 (0.0%)	7 (3.8%)	5 (6.3%)	<0.001
Primary/rev	185 (87.3%)	77 (97.5%)	130 (72.2%)	55 (69.6%)	<0.001

ABMS = anterior-based muscle-sparing, BMI = body mass index, CKD = chronic kidney disease, DAA = direct anterior approach, HTN = hypertension, MMH = modified Müller-Hardinge

There were 447 primary surgeries and 103 revision surgeries. Surgical approaches included 79 DAA (14%), 212 ABMS (39%), 180 MMH (33%), and 79 PL (14%). Anesthesia included 42 general (8%), 4 general + spinal (1%), 337 general + periarticular injection (61%), 161 spinal + periarticular injection (29%), and 6 with general + spinal + periarticular injection (1%). The patients were significantly different at baseline among the four approaches for average age (*P* < 0.001), BMI (*P* < 0.001), smoking history (*P* = 0.014), use of blood thinners (*P* < 0.001), and percentage of primary surgeries (*P* < 0.001). Information regarding the surgeons included in this study is summarized in Table 2. Statistical analysis of primary cases alone and revision cases alone showed no differences compared with the analysis of the entire cohort, so we have reported the results of the cohort as a whole. Table 3 presents the indications for primary arthroplasty, and Table 4 presents the indications for the 103 revision surgeries. The number of primary osteoarthritis cases per surgeon relative to the total number of cases per surgeon is summarized in Table 5.

**Table 2:** Case Numbers and Experience Levels per Surgeon

Surgeon	Cases	THA Approach(es)	THA Type	Experience Level (yrs)	LOO (min)	LOS (d)	BL (mL)	Complications Excluding LFCN	LFCN Injuries
1	179	176 MMH, 3 PL	131 PRI, 48 REV	>15	153	2	350	14	0
2	113	78 DAA, 35 PL	99 PRI, 14 REV	>15	141	1	335	15	6
3	22	22 PL	17 PRI, 5 REV	0-5	165	2	318	0	0
4	217	212 ABMS, 1 DAA, 4 MMH	186 PRI, 31 REV	>15	97	2	322	17	0
5	19	19 PL	14 PRI, 5 REV	0-5	139	1	361	0	0

ABMS = anterior-based muscle-sparing, BL = blood loss, DAA = direct anterior approach, LFCN = lateral femoral cutaneous nerve, LOO = length of operation, LOS = length of hospital stay, MMH = modified Müller-Hardinge, PL = posterolateral, THA = total hip arthroplasty

**Table 3:** Indications for Primary Arthroplasty

Indications for Primary Arthroplasty	No. of Cases	Percentage
Acute femoral neck fracture	1	0.2
Acute hip fracture	16	3.6
Osteonecrosis	26	5.8
Childhood hip problem (eg, hip dysplasia)	9	2.0
Degenerative arthritis (eg, OA)	358	80.1
Inflammatory arthritis (eg, RA, AS, and SLE)	7	1.6
Hip fracture (eg, nonunion and hardware failure)	11	2.5
Posttraumatic arthritis	18	4.0
Tumor (joint metastasis)	1	0.2
Total no. of primaries	447	100

OA = osteoarthritis

**Table 4:** Indications for Revision Arthroplasty

Indications for Revision Arthroplasty	No. of Cases	Percentage
Adverse local tissue reaction	6	5.8
Aseptic loosening	23	22.3
Bearing weight (eg, poly wear)	6	5.8
Infection	27	26.2
Instability	21	20.4
Osteolysis (including reaction to metal debris)	8	7.8
Periprosthetic fracture	12	11.7
Total no. of revisions	103	100

**Table 5:** Number of Cases of Primary Osteoarthritis (OA) and Total Cases per Surgeon

Surgeon	OA Cases/Total Cases (%)
1	108/179 (60.3)
2	92/113 (81.4)
3	11/22 (50.0)
4	135/217 (62.2)
5	12/19 (63.2)

Overall, there were 53 complications in 52 patients (9.5%), summarized in Table 6. A significant difference was observed among the four approaches for the probability of complications (*P* = 0.008) after adjusting for age, BMI, smoking history, preoperative blood thinners, primary versus revision, and chronic conditions. This finding is driven by the comparisons between ABMS and MMH versus DAA, and MMH versus POST. The odds of complications were 2.9 times greater for DAA versus ABMS (95% CI, 1.29-6.62), 13.4 times greater for DAA versus MMH (95% CI, 2.56-69.83), and 5.8 times greater for POST versus MMH (95% CI, 1.31-25.64). The difference in complication rates was not significant if the lateral femoral cutaneous nerve (LFCN) injuries were excluded from the analysis (*P* = 0.13).

**Table 6:** Type of Complication by THA Approach

THA Approach Overall Rate	ABMS	DAA	MMH	PL	Total
Deep infection (PJI)					
Primary	0/185	0/77	2/130	1/55	3/447
Revision	1/27	0/2	2/50	0/24	3/103
Superficial infection (cellulitis)					
Primary	3/185	0/77	0/130	0/55	3/447
Revision	0/27	0/2	0/50	0/24	0/103
Dislocation					
Primary	1/185	4/77	1/130	1/55	7/447
Revision	0/27	0/2	0/50	2/24	2/103
Periprosthetic fracture					
Primary	4/185	0/77	2/130	0/55	6/447
Revision	0/27	0/2	3/50	0/24	3/103
Nerve injury					
Primary	1/185 (sacral plexopathy)	7/77 (6 lateral cut fem n., 1 SN)	0/50	1/24 (1 deep peroneal n.)	9/447
Revision	1/27 (femoral n.)	0/2	1/50	3/24 (1 femoral n., 1 common peroneal n., 1 sciatic n.)	4/103
Thigh hematoma (I&D)					
Primary	3/185	0/77	0/130	0/55	3/447
Revision	0/27	0/2	0/50	0/24	0/103
Wound dehiscence					
Primary	0/185	0/77	1/130	1/55	2/447
Revision	0/27	0/2	2/50	0/24	2/103
Mechanical complication					
Primary	0/185	0/77	0/130	0/55	0/447
Revision	0/27	0/2	1/50 (acetabular loosening)	1/24 (femoral loosening)	2/103
Medical complication					
Primary	2/185 (1 PE/DVT, 1 asp pneumonia)	0/77	0/130	0/55	2/447
Revision	0/27	0/2	1/50 (excision HO)	1/24 (1 PE/DVT)	2/103
Complication rate	16/212 (7.5%)	11/79 (13.9%)	16/180 (8.9%)	11/79 (13.9%)	

ABMS = anterior-based muscle-sparing, DAA = direct anterior approach, deep-vein thrombosis, HO = heterotrophic ossification, MMH = modified Müller-Hardinge, PE = pulmonary embolism, PL = posterolateral, THA = total hip arthroplasty

The most common complication was nerve injury (13, 2.4%) followed by periprosthetic fracture (9, 1.6%), THA dislocation (9, 1.6%), periprosthetic joint infection (6, 1.1%), wound dehiscence (4, 0.7%), superficial infection (3, 0.5%), thigh hematoma requiring irrigation and débridement (3, 0.5%), deep-vein thrombosis or pulmonary embolism (2, 0.4%), and others (3, 0.5%). Other complications included aspiration pneumonia (1, 0.2%) in ABMS, acetabular loosening (1, 0.2%) in MMH, and femoral loosening (1, 0.2%) in the PL approach. The subgroup of dislocations in the DAA group was examined more closely. Two of those patients had frank dislocations, and two were identified as “unstable” because of subluxation as noted by the treating surgeon. One of the patients with subluxation was revised to a constrained liner, and one was treated nonsurgically. The two that dislocated were both revised. One was noted to have generalized ligamentous laxity, and the other did not have a diagnosed reason for the dislocation. One patient in the MMH cohort had multiple complications, including a MRSE PJI and symptomatic heterotrophic ossification requiring excision. At the 6-month follow-up, 20 patients had asymptomatic heterotrophic ossification detected on radiographic evaluations. In addition, 15 presented with mild trochanteric bursitis.

Estimated blood loss was highly skewed; therefore, the natural log transformation was applied to improve the approximate normality for the analysis. No significant differences were observed among the approaches in average blood loss (natural log transformed, *P* = 0.51) or having one or more transfusion units (*P* = 0.26) after adjusting for age, BMI, preoperative blood thinners, primary versus revision, and chronic conditions. The average model-adjusted EBL (geometric mean) was 326.0 mL (coefficient of variation 0.55) and ranged across the approaches from 284.7 for ABMS to 374.9 for DAA. Three hundred fifty-seven patients received no blood transfusion, and 193 patients received one or more units postoperatively. Of the 193 who did receive blood, the median transfusion amount was 2.0 units (range, 1-9 units). Table 7 presents the median transfusion unit by approach.

**Table 7:** Median Units of Transfusion by THA Approach

THA Approach	No. With Transfusion (%)	Median Units of Transfusion (Min, Max)
ABMS	61 (29)	2 (1, 8)
DAA	30 (38)	2 (1, 3)
MMH	71 (39)	2 (1, 9)
POST	31 (39)	2 (1, 8)
Total no. of patients with transfusions	193 (35)	2 (1, 9)

ABMS = anterior-based muscle-sparing, DAA = direct anterior approach, MMH = modified Müller-Hardinge, THA = total hip arthroplasty

Table 8 presents the model-adjusted means and 95% CI of the HOOS, UCLA, HHS, and VR Mental/Physical clinical outcome scores regarding the THA approach. A statistically significant difference was observed in the average UCLA change (preoperative versus 1 year) among the four approaches (*P* = 0.020) adjusted for age, BMI, preoperative blood thinners, primary versus revision, and chronic conditions. This was driven by the comparisons between ABMS and DAA versus POST (*P* = 0.012 and *P* = 0.003, respectively).

**Table 8:** Model-Adjusted Means and 95% Confidence Intervals of the HOOS, UCLA, HHS, and VR Mental/Physical Clinical Outcome Scores Regarding THA Approach

Clinical Outcome Score	Model-Adjusted Mean	95% Confidence Limits	*P* Value
HOOS change (n = 304)			0.79
ABMS	31.72	20.92 to 42.52	
DAA	33.21	21.17 to 45.26	
MMH	24.58	13.25 to 35.91	
POST	34.06	22.27 to 45.84	
UCLA change (n = 304)			**0.02**
ABMS	1.89	0.94 to 2.85	
DAA	2.21	1.14 to 3.29	
MMH	0.98	−0.03 to 1.99	
POST	1.05	0.002 to 2.10	
HHS change (n = 272)			0.43
ABMS	34.84	23.99 to 45.70	
DAA	37.78	25.23 to 50.33	
MMH	22.86	11.44 to 34.28	
POST	38.75	26.55 to 50.95	
VR mental change (n = 305)			0.43
ABMS	5.25	−0.76 to 11.25	
DAA	2.04	−4.71 to 8.79	
MMH	2.24	−4.11 to 8.60	
POST	4.84	−1.74 to 11.41	
VR physical change (n = 305)			0.49
ABMS	14.64	8.67 to 20.62	
DAA	16.98	10.26 to 23.69	
MMH	9.21	2.89 to 15.53	
POST	14.55	8.01 to 21.09	

ABMS = anterior-based muscle-sparing, DAA = direct anterior approach, HHS = Harris Hip Score, HOOS = Hip Disability and Osteoarthritis Outcome Score, MMH = modified Müller-Hardinge, THA = total hip arthroplasty

The LOO differed among the four approaches (natural log transformed for analysis, *P* = 0.042). The ABMS approach averaged 43.5 minutes less than MMH (*P* = 0.017), which was statistically significant after adjusting for age, BMI, preoperative blood thinners, primary versus revision, and chronic conditions (geometric means 117.4 versus 160.9 minutes).

A significant difference was observed among the 4 approaches for the probability of LOS ≥2 days (*P* = 0.038) after adjusting for age, BMI, preoperative blood thinners, primary versus revision, and chronic conditions. The odds of an LOS ≥2 days were 3.9 times greater for ABMS versus MMH (95% CI, 1.18-12.92) and 4.4 times greater for POST versus MMH (95% CI, 1.32-14.71). However, this finding may be related to some initiatives aimed at shortening hospital stay that might have applied to one surgeon more than the others during the period studied. The retrospective nature of this study precludes further elucidating this effect.

## Discussion

Both in the United States and worldwide, the PL approach is the most popular approach for primary and revision THA.^20^ Despite providing excellent anatomic exposure to the proximal femur and acetabulum, some have argued that this approach is prone to higher rates of dislocation^4,21^ and sciatic nerve injury.^7^ Approximately 42% of the orthopaedic surgeons worldwide use the direct lateral or MMH approach.^20^ Advocates of the MMH approach report lower rates of dislocation compared with posterior approaches.^21,22^ However, critics suggest that the muscle-splitting lateral approach leads to greater postoperative pain, abductor weakness, prolonged hospitalization times, and more extensive rehabilitation.^4,23,24^ Proponents of the DAA believe that it demonstrates more rapid recovery, less postoperative pain, lower dislocation rates, and better functional outcomes compared with the transgluteal approaches.^24-26^ However, the DAA has also been associated with higher rates of intraoperative femoral fracture, wound complications, and injury to the LFCN.^6,25,27^ Purported advantages of the ABMS approach include supine positioning, muscle-sparing approach, preservation of the posterior capsule, low incidence of LFCN injury,^5,9,10,15,28,29^ revisions, and ability to extend the exposure for revisions.^18^ Some suggest that greater trochanter fracture^10,16^ and implant subsidence^30^ are more likely to occur with the ABMS approach.

The results of our study are consistent with other studies that show the surgical approach is not an independent predictor of outcome in the early postoperative period for primary THA.^16,30,31,32,33^ Although fewer in number, our study also includes the results of revision procedures demonstrating that the ABMS approach can be successfully applied to revision arthroplasty. We are aware of only one other report describing the use of the ABMS approach for revision surgery.^18^ Shigemura et al^18^ reported two patients who had developed polyethylene liner wear years after their primary THA through the MMH and PL approaches, respectively. The ABMS approach was used for both revision surgeries. At the 2-year follow-up, they reported successful functional capacity and radiographic evaluation.

In our series, we found a notable difference in perioperative complications but not in-hospital findings or clinical outcome measures among the ABMS, DAA, MMH, and PL approaches. ABMS and MMH had fewer complications than DAA, and MMH had fewer complications than POST. However, this statistical difference seems to be related to the number of LFCN injuries in the DAA patients. In the absence of the LFCN injuries, no statistically significant difference was observed in complication rates. Despite being done in the supine position, the more posterior location of the ABMS incision relative to the DAA incision likely explains the lack of LFCN injuries in that cohort compared with the DAA. Although we did find statistically better UCLA scores for the DAA versus other approaches, the difference is small and not supported by the other reported outcome scores (HOOS, HHS, and VR-12).

Given that there were a limited number of surgeons in this study, differences reported relative to the surgical time are likely surgeon-specific and cannot be generalized. Although we also report shorter LOS in the DAA group, we are cautious in our interpretation of this finding because we could not exclude the effect of individual surgeon preference on this metric.

Limitations of this study include all those inherent in retrospective reviews relative to fidelity and accuracy of the electronic medical record. Radiographic parameters, such as cup position, could not be reliable and compared because of the lack of standardized radiograph protocols for all patients. The results from a single-institution study and select group of surgeons, such as this one, cannot necessarily be applied to all institutions and surgeons. Owing to the low number of surgeons included in this study, factors such as LOS and LOO may also be affected by intangible factors, such as individual surgeon preferences and protocols, which cannot be completely accounted for in this study. The sample size may be too small to detect subtle differences in rare complication rates.

In a similar study of primary arthroplasty, Greidanus et al^30^ found that outcomes using the ABMS approach did not differ from outcomes from PL, MMH, and DAA approaches. Klassen et al^31^ compared the DAA with the ABMS approach and found no difference in total blood loss but also reported greater rates of dislocation and lower rates of transfusion, superficial infections, and overall complications in the DAA compared with the ABMS cohort. Comparing supine ABMS with the DL approach, George et al^5^ reported markedly lower opioid consumption during postoperative days 1 and 2 and 1.91 times less likelihood of experiencing postoperative abductor insufficiency in the ABMS cohort relative to the MMH. Kagan et al^11^ found no notable differences in surgical time, EBL, or immediate postoperative complications but did find slightly shorter LOS for the ABMS approach compared with the PL approach. They reported no difference in the adjusted mean change in patient-reported outcomes. Civinini et al^10^ reported lower complication rates and faster recovery in the first 6 weeks using the ABMS approach compared with the DAA. D'Arrigo et al^28^ found lower complication rates in the ABMS group compared with MMH and DAA and also reported improved early functional scores in both ABMS and DAA compared with MMH. Martin et al^32^ reported no difference in patient-reported outcomes 1 year postoperatively between ABMS and MMH approach groups.

## Conclusion

For primary and revision THA, we found no notable differences in perioperative complications, except for the incidence of LFCN injury. We found no notable difference in in-hospital findings or clinical outcome measures among the ABMS, DAA, MMH, and PL approaches. UCLA outcome scores were slightly better for the DAA patients, but those differences were not seen with HOOS, HHS, or VR-12 outcome measures. The supine ABMS approach seems to be a safe and effective option for THA. In the hands of surgeons well-versed and experienced with the ABMS results equivalent to other common THA approaches can be achieved.
